# Bilateral Peripheral Nerve Field Stimulation for Intractable Coccygeal Pain: A Case Study Using Dual Lead Intercommunication

**DOI:** 10.7759/cureus.1832

**Published:** 2017-11-09

**Authors:** Michelle Granville, Patrick T Brennan, Robert E Jacobson

**Affiliations:** 1 Miami Neurosurgical Center, University of Miami Hospital; 2 Pain Management, Larkin Community Hospital

**Keywords:** coccygeal pain, peripheral field stimulation, sacral pain, peripheral nerve field stimulation, post laminectomy pain, sacroplasty, dual lead communication

## Abstract

Coccygeal pain is a difficult chronic pain problem with mixed response to various treatments. This is a report of a case of coccygeal pain that after failing various conservative and interventional procedures over five years was evaluated with a temporary peripheral sacral fascial lead followed by implantation of bilateral sacral paramedian leads for peripheral nerve field stimulation (PNFS). This resulted in marked pain control and resumption of full activity. The visual analog scale (VAS) pain score improved from eight pre-implant to one after implant and has remained at that level in follow-up. Peripheral nerve field stimulation has been reported for axial chronic back pain, post-laminectomy pain and sacroiliac joint pain either alone or in conjunction with epidural spinal cord stimulation. Both single and parallel leads have been used to provide wider stimulation but differences in location have not been examined. This is the first case report of the use of PNFS for treatment of intractable chronic coccygeal pain. The effectiveness of PNFS was established for this patient by using a prolonged 10-day temporary trial period followed by a 30-day interval without stimulation during which the pain returned to the pre-trial level before proceeding with permanent implantation, it was clear that in this case, PNFS was effective for pain control. Interestingly, the trial and permanent leads were both in the posterior sacral fascia but not in identical positions yet equally effective for pain control. The observation of the effectiveness of different positions may indicate that at least for peripheral field stimulation there may be significant current spread in the fascia. Two and three months after the implant, we examined the effect of different lead settings and the effect of unilateral stimulation compared with bilateral stimulation with and without interlead communication. The patient feedback in this case provides some understanding of the effect of field stimulation with different lead placements. A trial of a deep peripheral fascial lead for sacral and coccygeal field stimulation is a simple option and may be a reasonable approach to consider in the range of treatments for chronic coccygeal pain.

## Introduction

Coccygeal pain remains a difficult problem. The etiology of the pain often is unclear and treatments not consistently effective [1]. Treatment options range from physical therapy to localized injections, ganglion impar blocks and rarely coccygectomy [1,2]. Recurrent and chronic cases can undergo radiofrequency ablation of the sacral sensory branches. Spinal cord and even intra-sacral stimulation has been tried but overall results are poor [3]. In cases of sacral coccygeal fractures use of bone cement may stabilize the fracture although the use in pure coccygeal fractures without associated sacral fractures has had mixed results. Peripheral nerve field stimulation (PNFS) using electrical impulses in the fascia rather than the epidural space or directly on a nerve bundle has been used successfully for peripheral nerve pain, intercostal pain, axial back and post-laminectomy pain [4,5]. The use in patients with post-laminectomy pain has had mixed results because of difficulty getting the electrode in dense para-spinal scar and uncertainty regarding if the electrode should be placed paramedian and vertical or horizontal across the incision and midline [6]. Positioning parallel electrodes is necessary to create interlead communication which broadens the stimulation field, however, the exact distance between leads that will still allow cross lead communication has not been established [7,8]. This case reviews the use of PNFS for chronic previously intractable coccygeal pain. Viewed in the context of the reported experiences with PNFS, this case study is a step in broadening the understanding of the physiologic effect and possible uses of PNFS [7,9]. This case also highlights the importance of a significant long trial period before consideration of permanent implantation since these patients often undergo multiple procedures with only short-term pain control.

## Case presentation

The patient is a 66-year-old female with over five years of progressively worsening sacroiliac and coccygeal pain that had become constant and made the patient unable to sit for extended periods of time, and she stated the pain was worse later in the day. She was not able to sit directly on the right buttock. The patient had several falls in the past but the patient described that the pain started after bariatric surgery. She developed pain radiating both into the coccyx, right lower buttock and rarely to the posterior thigh. Magnetic resonance imaging (MRI) of the lumbar spine showed minimal facet joint spondylosis without any signs of herniated disc, nerve root or canal compression. The patient had failed or had short-term relief of less than 30 days from other conventional therapies for coccygeal pain including steroid blocks, ganglion impar blocks and radiofrequency rhizotomy of the sacroiliac joint. Radiographs, bone scan and computerized tomography (CT) scans showed an old coccygeal and possibly osteoporotic sacral fracture to the right. To determine the role the sacral insufficiency fracture played in causing her pain, four years after the pain started, she underwent injection of bone cement in both sacral alae and the coccyx. This resulted in the loss of sacroiliac joint pain but not her coccygeal pain. Six months later, she then had a trial with a thoracic spinal cord lead to T8 to T10 that had no effect on the coccygeal pain. She noted her pain as an eight to nine on visual analog scale (VAS) and was taking Oxycodone 5/325 mg from two to four times daily and gabapentin 300 mg four times daily. It was proposed to the patient to do a lower sacral trial stimulator implant as well as a subcutaneous sacral implant to evaluate if these areas would control her pain.

The trial procedure was performed under local anesthesia with 1% lidocaine and a trial eight-point percutaneous lead was positioned in the posterior epidural intra-sacral space through a 14 gauge Tuohy needle. The percutaneous lead (Medtronic, Minneapolis, MN) was positioned in the lower sacrum below the S2 level. In this location when the epidural lead was stimulated, the patient only felt tingling in the right buttock and minimal perineal tingling. There was absolutely no coccygeal stimulation which was the area of her primary pain. This lead was removed since stimulation was not obtained in the lower sacral or coccygeal area. Next, another eight-point lead was placed into the posterior deep sacral fascia from her more symptomatic right side. The lead was passed through a Tuohy needle from the inferior right para-sacral area towards the midline near S1. Trial stimulation gave the patient immediate tingling sensation bilaterally into the sacral and coccyx area exactly where her pain was located. The lead was sutured and taped in place. Further testing in the recovery room demonstrated strong stimulation bilaterally in the lower sacrum, medial buttocks and especially the coccyx. She went home and underwent an initial five-day trial, with daily contact by the office nurse on her progress.

**Figure 1:**
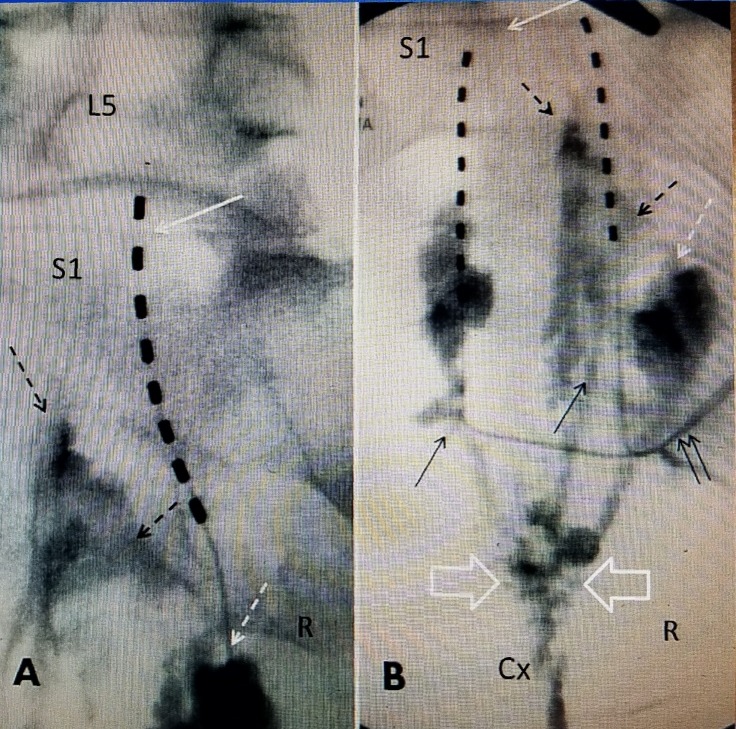
Composite picture of temporary trial lead, two permanent peripheral leads. (A) Temporary deep fascial lead placed from inferior right toward the midline upper sacrum. The lead starts paramedian and goes toward the midline. The top of sacrum is noted under fluoroscopy (solid white arrow). The previously placed sacroplasty cement for sacro-coccygeal fracture (dotted black) and sacral cement is noted (dashed white arrow). (B) Anterior-posterior intraoperative film showing two paramedian sacral peripheral field electrodes. The black arrows show path of each electrode passed from inferior to superior. The two solid arrows show electrodes tunneled together to the generator. The cement in the coccyx is noted at Cx. The coccygeal cement (open white arrows) is seen well below the entry point of the deep fascial electrodes that provide field stimulation in the paramedian sacral area. The dotted black arrow shows cement in the alae of the sacrum and sacroplasty cement with a dashed white arrow.

During the initial five-day trial, she continued with the same stimulation which provided coverage to the bilateral paramedian and lower sacrum and coccyx areas with marked pain relief. Her VAS score went from pre-procedure of eight to zero to one, there was a total change in facial expression and she spontaneously stopped all pain medication. When she returned to the office five days post-trial implant, rather than removing the lead, it was decided after discussion with the patient, to continue with the trial for another five days in an effort to ensure the relief continued while the patient resumed full activity. Over a total of 10 days trial period, the patient did her normal activities including taking care of special needs of grandchild and she continued with over 90% pain relief. She returned to the office and the temporary trial lead was then removed. After reviewing information on PNFS with the patient, it was proposed to proceed to do bilateral peripheral field electrode implantation explaining that the two leads would allow for stronger stimulation and coverage of the painful area. She had a scheduled trip so the permanent implant was actually performed almost 30 days after the initial trial terminated. During the 30-day period without the stimulator, she stated her pain completely returned to the pre-trial stimulation level with a VAS between eight and nine and she resumed taking hydrocodone 5/325 mg. She then had permanent implantation of two eight-point leads attached to a rechargeable Medtronic battery (Medtronic). Under fluoroscopic guidance, the permanent leads were placed bilaterally in a para-median direction on both sides of the lower sacrum so they were roughly parallel to each other. Repeat intra-operative testing of each lead demonstrated strong stimulation to the lower sacrum and midline coccyx regions. Both leads were in the deep posterior sacral fascia. Neither lead was adjacent to or making direct contact with bone in the coccyx or lower sacral area (Figure 1).

**Figure 2:**
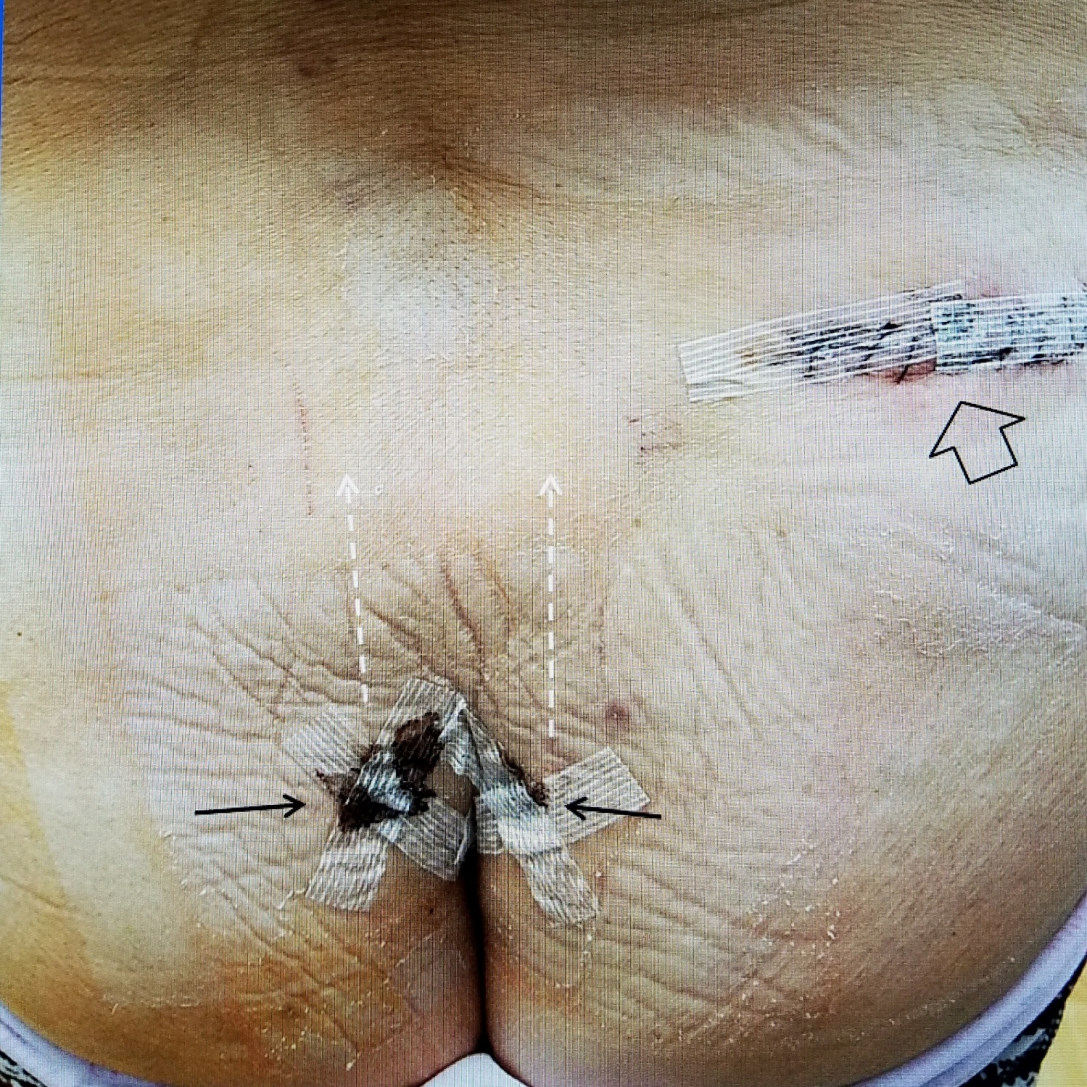
Picture of incisions for stimulator wires and permanent battery. Postoperative film five days after implant: The skin insertions for the leads are paramedian (solid black arrows) and direct the stimulator leads paramedian (dashed white arrows). The leads are tunneled to the generator in the upper right buttock (hollow black arrow).

The final implantation was performed under local anesthesia with mild sedation. The leads were passed together to the battery. At the time of implantation, both leads were separately tested and the patient felt the stimulation equally into the lower sacrum and coccygeal area (Figure 2).

The patient was seen at one, four, nine weeks and three months post-implant and remained with a VAS score of one, off all medications and fully active. She stated she was using the stimulator between two and four hours daily, primarily in the late afternoon and early evening. In attempting to better understand the effect of electrode position on peripheral field stimulation, post-implant testing of the different effects of unilateral stimulation and bilateral stimulation with and without inter-communication or 'cross-talk' between the leads was performed starting nine weeks after permanent implantation. At the time of post-implant testing, the patient had absolutely no incisional pain at either the lead or battery site, was very familiar with the stimulator sensation she received so she could concentrate on giving clear feedback on different sensations and the precise location of the stimulation with changes in stimulator settings.

The original post-implant settings for the permanent implant were the following which provided excellent pain relief for the initial nine weeks: four electrodes on each side with the following settings of left lead +2 and +3 and -5 and -6 and the right lead +10 and +11 and -13 and -14. At these settings, the patient felt equal stimulation bilaterally in the sacral para-spinal region midway between the iliac crest and down to the coccyx. The positive and negative electrodes were on the same lead and there was no intercommunication between the two parallel electrodes. At the follow-up office visit at nine weeks, each lead was tested separately, first at both the original settings and then making all eight points active with four points positive and four negative. Using only a unilateral lead, the patient felt stimulation primarily unilateral but also into the coccyx. There was minimum difference in tingling using four versus eight stimulation points on one side. Next, the leads were set to communicate and 'cross-talk' right to left, so the positive and negative were opposite each other in the two parallel leads. With this right to left intercommunication setting the patient clearly felt stronger midline and also coccygeal stimulation and was able to get pain relief with lower voltage settings compared to a single lead. She preferred this setting and was sent home to evaluate the difference in pain control. She returned in four weeks, which was 13 weeks after implant and stated that the pain was minimal, with a VAS between zero and one. She preferred the 'cross-talk' between the two leads which gave her greater 'coverage' across the lower midline sacrum. With stimulation, she was able to sit comfortably without the constant right buttock pain which had been making it difficult to sit without keeping her buttock off the chair.

## Discussion

The use of PNFS, also known as subcutaneous field stimulation, for sacral and coccygeal pain has not been previously reported. There are reports of using PNFS for iliac crest and sacroiliac pain with single and dual electrodes [4,8,9]. Nerve field stimulation evolved from the original concept of localized stimulation targeting specific branches of peripheral nerves. This technique was originally developed for pain control after peripheral nerve injuries and reflex sympathetic dystrophy in patients unresponsive to medication or spinal cord stimulation [4]. Subcutaneous electrodes have been implanted for different regional pain syndromes including post-herpetic pain, intercostal pain, hip and knee pain, trochanteric bursitis, post-joint replacement pain and for pain at iliac bone graft sites [4,9]. Some authors have used ultrasound guidance to position the electrodes in the deep fascia but in this case all leads were placed under fluoroscopic guidance [4,7,8]. There are both individual case reports and small series of patients that show significant relief using peripheral nerve or field stimulation for intractable axial lumbar pain as well as for post-laminectomy pain [4-7]. Use of PNFS alone without spinal cord epidural stimulation has been reported in patients with primarily axial lumbar pain. In these cases, the electrodes have been placed either vertically, in a paramedian position, along the length of the laminectomy incision or coronally across the laminectomy incision [6,7]. PNFS has also been combined with spinal cord stimulation to target axial postsurgical back pain when the patient also has more lateralized pain at an iliac crest graft site or along the greater trochanter for hip pain that cannot be reached with an intraspinal lead [10]. Dual parallel subcutaneous leads give a broader area of stimulation [7,8]. When the leads are made to communicate by programming different polarity at the same level this creates a current between the parallel leads, which has been labeled as 'cross-talk'. Physiologically, the current appears to spread within the subcutaneous tissue and fascia by intercommunicating between positive and negative polarity of two different leads rather than along the same lead [7]. The distance that this intercommunication can bridge depends on the tissue, the existence of surgical fibrosis and surgical scar [7,9,10]. In the largest series of 10 patients with axial post-laminectomy pain using dual parallel leads, six went on to permanent implant but only averaged about 45% reduction in pain after permanent implantation [6]. Follow-up studies found that pain relief was maintained through a 12-month period in patients that had both successful trial and relief from implantation [9]. Several reports have tried to equate the use of peripheral transcutaneous neural stimulation (TNS) to an implanted subcutaneous electrode trial but results and predictability of the effectiveness for a permanent implant have not been consistently based on patient response to TNS [4,9]. All reports have emphasized the importance of an effective temporary implant trial before consideration of permanent implantation similar to this case of coccygeal pain [4-9].

In this case where PNFS was used to treat intractable coccygeal pain, the trial and permanent leads were not in identical positions but the patient had equally strong stimulation and pain relief. The unilateral trial lead went from the inferior right buttock toward the midline over S1-S2, while the bilateral permanent leads were positioned at a slightly lower level but roughly parallel in a paramedian position with approximately 5 cm separating the leads. Neither lead was specifically over the painful area in the lower sacrum or coccyx. Post-procedure testing documented a very localized effect of each lead alone but when the two separate leads were made to communicate with each other there was definitely wider spread of the stimulation across the midline in the area between the leads as well as down towards the coccyx. The patient felt stronger midline and coccygeal sensation with dual lead cross stimulation which she preferred.

Communication or 'cross-talk' occurs between two parallel leads when there is opposite polarity across the subcutaneous tissue. It is not clear how great a distance can separate the leads but studies show widespread of current in normal subcutaneous tissue [7]. There are no reported studies on the difference of effect of electrical stimulation across scar compared to normal fascial tissue. Peripheral leads placed for post-laminectomy and axial pain have been positioned as far as 7 to 10 cm apart across the incision and separating vertically by two to three spinal segments and still were able to create cross-stimulation. It is postulated that by creating inter-lead communication there is both a stronger and a larger electrical field [7]. In a case study using PNFS for unilateral sacroiliac pain, two parallel leads, approximately 2 cm apart, were placed along the sacroiliac joint allowing the leads to 'communicate' which created strong and lasting pain relief [8]. The physiologic effect of field stimulation is obviously broader and less specific than what is seen with epidural spinal cord stimulation [4]. In this patient with coccygeal pain, while both sets of electrodes were placed at a similar sub-fascial depth over the posterior sacrum and not the coccyx, they were at least 5 cm apart but still able to cross stimulate. Each electrode separately stimulated and relieved her coccygeal pain but the two electrodes combined created a broader sensation with better pain relief at a lower voltage setting with some spread across the midline lower sacrum. It appears that properly positioning the lead in the general area is key to field stimulation since there are no specific nerve branches involved in the lower sacrum and coccygeal area.

## Conclusions

Chronic coccygeal pain is a difficult problem to manage if more conservative treatments using physical therapy, medications or localized blocks fail. After evaluating the patient's pain response using a temporary trial of a PNFS, it was possible to percutaneously place two electrodes in the lower posterior sacral region to provide nondestructive pain management in a patient with chronic coccygeal pain. The slightly different positions of the trial electrode lead and the two permanent leads highlight the variability and give some understanding of the current spread and effect of peripheral nerve field stimulation. The post-procedure testing in this case demonstrated that there are distinct differences in the area of stimulation using single versus dual leads. When the patient had dual lead stimulation there was clearly a broader sensation across the entire lower lumbar and sacral area requiring less power settings for pain relief. This case shows that PNFS could be another nondestructive option for pain management for coccygeal pain. Using a sub-fascial lead placed in the lower sacral area is a simple minimally invasive approach to manage coccygeal pain.
